# Draft Genome Sequence of *Coxiella burnetii* Historical Strain Leningrad-2, Isolated from Blood of a Patient with Acute Q Fever in Saint Petersburg, Russia

**DOI:** 10.1128/genomeA.01464-17

**Published:** 2018-01-18

**Authors:** Olga Freylikhman, Artem Kiselev, Sergey Kazakov, Alexey Sergushichev, Yulia Panferova, Nikolay Tokarevich, Anna Kostareva

**Affiliations:** a Saint-Petersburg Pasteur Institute, Saint Petersburg, Russia; b Almazov National Medical Research Centre, Saint Petersburg, Russia; c ITMO University, Saint Petersburg, Russia; d JetBrains Research, Saint Petersburg, Russia

## Abstract

This is the announcement of a draft genome sequence of *Coxiella burnetii* strain Leningrad-2, phase I. The strain, which is mildly virulent in infected guinea pigs, was isolated in 1957 from the blood of a patient with acute Q fever in Leningrad (now Saint Petersburg), Russia.

## GENOME ANNOUNCEMENT

*Coxiella burnetii* is a strictly intracellular bacterium and the etiological agent of Q fever. Infection in humans is characterized by pronounced polymorphism of disease manifestations without pathognomonic symptoms. Moreover, the clinical presentations of *C. burnetii* infection depend on both the strain virulence and specific risk factors associated with the patient (1).

We studied the *C. burnetii* strain, named Leningrad-2, which had been isolated in 1957 in Leningrad (now Saint Petersburg), Russia, from blood of a 42-year-old female patient with acute Q fever during an outbreak recorded among workers at a production facility. The course of disease with symptoms of pneumonia was benign, and the patient recovered in 8 to 10 days.

The outbreak took place at a cotton-processing facility and included 58 serologically confirmed cases. All patients were workers engaged in the processing of raw cotton brought from central Asia, where Q fever was then enzootic. Despite the same mechanism of infection, clinical manifestations of the disease in patients were dissimilar (2).

It is interesting that according to multispacer sequence typing (MST), strain Leningrad-2 is classified as sequence type 7, which is not typical for northwestern Russia. This sequence type also occurs very rarely all over the territory of the former USSR, where the MST23 genotype certainly dominates (3).

The Leningrad-2 strain is serologically in phase I (4) and proved to be mildly virulent in infected guinea pigs (5, 6). The strain was grown in the yolk sacs of chicken embryos (7, 8), and DNA was isolated using the QIAamp DNA minikit (Qiagen, USA).

The whole-genome sequencing of this *C. burnetii* strain was performed using the Illumina MiSeq sequencing technology with the paired-end and barcode strategies, according to the manufacturer’s instructions (Illumina, USA).

*De novo* genome assembly was carried out with the SPAdes version 3.6.2 software (9). The genome coverage was 35.0×. We obtained 93 scaffolds, with an *N*_50_ of 42,843 bp and a largest scaffold length of 194,351 bp. The genome sequence of the Leningrad-2 strain contains 2,085,158 bp, with a G+C content of 42.60%.

The draft genome sequence was submitted to GenBank and annotated using the NCBI Prokaryotic Genome Annotation Pipeline (PGAP) (http://www.ncbi.nlm.nih.gov/genome/annotation_prok). PGAP found a total of 2,320 genes, 2,271 coding sequences (CDSs), and 208 pseudogenes. Leningrad-2 contains plasmid QpRS (38,538 bp), which is also not typical for the strains circulating in Russia that usually contain plasmid QpH1.

Thus, although host organism factors significantly influence *C. burnetii* pathogenicity realization, the analysis of new genomic data has enabled a description of the diversity of strains around the world and their link with pathogenicity.

### Accession number(s).

The annotated draft whole-genome sequences of the chromosome and QpRS plasmid of the *C. burnetii* Leningrad-2 human strain have been deposited in DDBJ/ENA/GenBank under the accession number PDLP00000000. The version described in this paper is version PDLP01000000.
